# Stability and Bioaccessibility of Carotenoids from Sea Buckthorn Pomace Encapsulated in Alginate Hydrogel Beads

**DOI:** 10.3390/nu16162726

**Published:** 2024-08-16

**Authors:** Cristina Elena Gherasim, Monica Focşan, Călina Ciont, Andrea Bunea, Dumitriţa Rugină, Adela Pintea

**Affiliations:** 1 Department of Chemistry and Biochemistry, University of Agricultural Sciences and Veterinary Medicine, Mănăştur Street 3–5, 400372 Cluj-Napoca, Romania; cristina.gherasim@usamvcluj.ro (C.E.G.); andrea.bunea@usamvcluj.ro (A.B.); apintea@usamvcluj.ro (A.P.); 2 Biomolecular Physics Department, Faculty of Physics, Babeș-Bolyai University, 1 Mihail Kogalniceanu Str., 400084 Cluj-Napoca, Romania; monica.iosin@ubbcluj.ro; 3 Nanobiophotonics and Laser Microspectroscopy Center, Interdisciplinary Research Institute on Bio-Nano-Sciences, Babeș-Bolyai University, Treboniu Laurean Street, 42, 400271 Cluj-Napoca, Romania; 4 Institute of Life Sciences, University of Agricultural Science and Veterinary Medicine, Calea Mănăstur 3–5, 400372 Cluj-Napoca, Romania; calina.ciont@usamvcluj.ro

**Keywords:** sea buckthorn, zeaxanthin, alginate hydrogel beads, stability, bioaccessibility

## Abstract

Carotenoids, the natural pigments that confer the bright orange color of sea buckthorn berries, are also associated with several health benefits, such as antioxidant activity and skin and eye protection. Due to their lipophilic nature and localization, carotenoids are largely retained in the sea buckthorn pomace (SBP) resulting from juice production. Carotenoids from SBP (70.03 mg/100 g DW), extracted and characterized by HPLC-PDA, contained zeaxanthin (free and esterified) and beta-carotene as major compounds. The SBP carotenoids-enriched sunflower oil was further encapsulated in Ca-alginate hydrogel beads (98.4% encapsulation efficiency) using ionotropic gelation. The hydrogel beads were characterized by confocal laser scanning microscopy and scanning electron microscopy. Fairly good stability (>64%) of the encapsulated carotenoids in the alginate hydrogel beads during storage (30 days, 4 °C and 25 °C) was found, with zeaxanthin esters being the most stable compounds, for all the experimental conditions. The bioaccessibility of the total carotenoids (INFOGEST protocol) was 42.1 ± 4.6% from hydrated, and, respectively, 40.8 ± 4% from dehydrated SBP alginate hydrogel beads. The addition of yogurt to the dehydrated hydrogel beads had a positive effect on the bioaccessibility of free and esterified zeaxanthin, but not on that of the carotenes. In conclusion, SBP is a valuable source of carotenoids which can be protected by encapsulation in alginate hydrogel beads, thus still retaining a good bioaccessibility.

## 1. Introduction

Sea buckthorn berries are classified as super fruits, due to their high content of major nutrients (lipids and essential fatty acids, carbohydrates, proteins) but also of vitamins, minerals, and lipophilic and hydrophilic bioactive compounds [1,2,3]. Sea buckthorn is extensively studied for its health benefits, such as its antioxidant, immunomodulatory, anti-diabetic, or anti-cancer effects [1,4,5]. Among lipophilic compounds, carotenoids stand as valuable constituents in SB, not only due to their provitamin A activity (e.g., β-carotene; β-cryptoxanthin), but also to the biological properties of non-provitamin A xanthophylls, zeaxanthin, and lutein. In addition to their role in photosynthesis in plant and algae, carotenoids are associated with a plethora of health effects in humans and animals, acting as antioxidants, modulators of the immune systems, and protecting against photooxidative damage, metabolic syndromes, and neurodegenerative diseases or cancer [6,7]. Despite a relatively large variability in the total content and profile of carotenoids, which is strongly influenced by genetic and environmental factors, sea buckthorn berries remain one of the most valuable sources of zeaxanthin and β-carotene for the human diet. Previous studies, including some performed by our group, underscored the high bioaccessibility of xanthophylls from sea buckthorn, as well as their antioxidant and antiproliferative properties [8,9,10]. Zeaxanthin is among the most relevant compounds sourced from sea buckthorn, known as one of the macular carotenoids, compounds which are effective in preventing age-related macular degeneration (AMD) [6,7,11]. Sea buckthorn pomace obtained after juice production still retains important amounts of bioactive compounds, especially carotenoids and tocopherols, which are highly concentrated in the peels, seeds, and remaining pulp. These compounds can be valorized through efficient and green extraction methods and incorporated into food, food supplements, or feed formula in order to provide health protective effects, following the principles of circular economy at the same time [12,13,14,15,16,17,18].

The limited solubility of carotenoids and their susceptibility to thermal or oxidative degradation represent challenges for their incorporation into food and pharmaceutical products [19]. Encapsulation methods have emerged as effective strategies to protect carotenoids from degradation, to improve their solubility, and enhance their bioaccessibility/ bioavailability. Several types of micro- or nano-encapsulation processes have been developed, depending on the encapsulated materials, particle properties, or the aim of the encapsulation, etc. Among them, one can mention emulsion-based delivery systems, liposomes, inclusion complexes (most commonly with cyclodextrins), solid lipid nanoparticles (SLNps), nanostructured lipid carriers (NLCs), polymeric micro/nanoparticles, etc. The encapsulation technologies offer protection of carotenoids against the external environment through a coating that acts as a physical barrier. Moreover, nano- and microencapsulation can boost the bioaccessibility of carotenoids and favor their controlled release [20,21,22,23]. Additionally, the use of natural polymers and biocompatible materials as wall materials for encapsulation ensures the safety and sustainability of these delivery systems. There are multiple types of micro- and nano-encapsulation methods, but the selection of the most suitable one depends on the carotenoid properties, the desired physical characteristics of the carrier (dimensions, stability, solubility, etc.), the purpose of encapsulation, and the cost of the encapsulation process [19,24,25,26,27]. Several types of ingredients and coatings were used to encapsulate SB-derived materials (oil, oleoresin, lipoproteins), including alginate alone or in combination with carrageenan [28,29,30,31,32] gum, Arabic and β-cyclodextrin [33], beta-glucan from barley [34], whey protein and acacia gum [35,36], or bile salt-based vesicles (bilosomes) [37]. In the present study, sodium alginate, generally regarded as a safe compound by the FDA (GRAS) and approved in the EU as E 401, was chosen as the coating agent for encapsulation of the SBP extract rich in carotenoids, based on its well documented properties like biodegradability, biocompatibility, gel controlling ability, and thermal and chemical stability [38,39]. Moreover, encapsulated carotenoids have been tested for their coloring and sensory properties, stability, and bioaccessibility in various food models, like yogurt, ice cream, mayonnaise, or cookies [30,31,40,41]. Among them, yogurt is representative for the functional food market, having the advantage of being widely consumed, being a good carrier for probiotic bacteria, as well as an efficient vehicle for delivering both hydrophilic and lipophilic bioactive compounds [42].

Considering all the aspects mentioned above, SBP emerged as a potential source of carotenoids, especially of zeaxanthin, which together with lutein, are dietary nutrients associated with a low risk of AMD. Opposite to lutein, which is abundant in green vegetables, zeaxanthin is less common and the ratio between the two pigments in the human diet is about 5:1, thus offering a rationale to find novel and rich sources of bioaccessible zeaxanthin [6,11]. However, due to their polyenic framework and hydrophobicity, carotenoids are prone to oxidation, to chemical degradation (acidic environment), and have relatively low bioavailability. In this context, the efficient extraction and the protection of carotenoids from SBP by encapsulation in alginate hydrogel beads is worth investigating, considering their use in food or nutraceutical applications.

The aims of the present study were: (i) to efficiently extract and characterize the carotenoid fraction from sea buckthorn pomace (SBP); (ii) to encapsulate the lipophilic extract from SBP in alginate hydrogel beads; (iii) to determine the stability of encapsulated carotenoids over time, in different conditions; and (iv) to determine the bioaccessibility of major carotenoids from the SBP alginate hydrogel beads alone and from yogurt samples supplemented with SBP alginate hydrogel beads. 

Although a number of studies reported the encapsulation of sea buckthorn extracts, our study includes novel information regarding the structure of the SBP alginate hydrogel beads (RCM, SEM) and the stability and the bioaccessibility of the total and major individual carotenoids (HPLC-PDA), depending on the type of hydrogel beads used (hydrated/dehydrated). Moreover, the assessments of the bioaccessibility of individual carotenoids from the SBP alginate hydrogel beads added to a food matrix (yogurt) is an original aspect. Last but not least, our study presents a way of valorizing a by-product (SBP), by using ecological extraction methods and affordable (sunflower oil) and safe (sodium alginate) materials. 

## 2. Materials and Methods

### 2.1. Plant Material 

In order to obtain the pomace, sea buckthorn berries (wild type) were processed with a low-speed juicer (Kuvings B1700, Kuvings-Romania NYS Experience S.R.L, Bucharest, Romania) and filtered. The pomace obtained after filtration contained the peel, seeds, and residual pulp. The pomace powder was obtained after drying for 48 h, at 39 °C (POL-EKO sp.k, Wodzisław Śląski, Poland) and grinding (Heinner HCG-200DGIX2, NOD S.R.L Bucharest, Romania). The obtained powder was stored in sealed aluminum coated polyethylene at −20 °C until further analysis. 

### 2.2. Chemicals and Standards

The enzymes used for the simulated in vitro digestion protocol were purchased from Sigma–Aldrich (Steinheim, Germany) including α-Amylase from human saliva (A1031), pepsin from porcine gastric mucosa (P6887), pancreatin from porcine pancreas (P7545), and bovine bile extract (B3883). Carotenoid standards including β-carotene, lycopene, β-cryptoxanthin, zeaxanthin, and zeaxanthin dipalmitate were purchased from Extrasynthese (Lyon, France). All the chemicals and reagents were of analytical or HPLC grade, purchased from Merck (Darmstadt, Germany) and Sigma–Aldrich (Steinheim, Germany). Ultrapure water was obtained using a Milli-Q water purification system. 

### 2.3. Carotenoid Extraction from SBP

Carotenoids were extracted from the SBP powder (50 g) using ultrasound-assisted extraction protocol and MeTHF as the extraction solvent (500 mL) as previously described [43]. After the solvent removal, the extracted carotenoids were dissolved in 80 g sunflower oil. For spectrophotometric and HPLC-PDA analysis, the sunflower oil SBP extract was dissolved in methyl-*tert*-butyl-ether (MTBE) and filtered through 0.22 μm PTFE filters. 

### 2.4. Quantitative Determination of Carotenoids Using Spectrophotometric Analysis

The spectrophotometric analysis was used in order to determine the total carotenoid content from samples, following the method described by Britton (1995) [44]. An UV-VIS Spectrophotometer (Jasco V-530, ABL&E-JASCO România S.R.L, Cluj-Napoca, Romania) was used and the total carotenoid content was expressed as mg carotenoids/100 g powder or alginate hydrogel beads, applying the following Equation (1):

(1) $$C=\frac{Abs\times V\times D\times 1000}{A_{1cm}^{1\%}\times 1\times 100}$$

where Abs means the absorbance read at 450 nm, V is the sample volume expressed in mL, A1% (2500) represents the absorption coefficient (1 cm), defined as the theoretical absorbance of a solution of 1% (*w*/*v*) concentration in a cuvette with 1 cm path length. 

### 2.5. Identification and Quantification of Carotenoids by HPLC-DAD 

Carotenoids separation from SBP and digests was performed on a YMC C30 reversed-phase column (250 × 4.6 mm i.d., 5 µm particle size), using an HPLC system (Shimadzu Corporation, Kyoto, Japan) equipped with a SPDM20A diode array detector and a gradient elution, as previously described [9]. Major carotenoid pigments were identified based on their retention times, absorption maxima, and spectral characteristics (% III/II), which were compared with those of available standards and literature data. Quantification of carotenoids was performed by external calibration method. Standard curves were built with β-carotene, lycopene, zeaxanthin, β-cryptoxanthin, and zeaxanthin dipalmitate in the range of 1–100 µg/mL.

### 2.6. Encapsulation of Carotenoid Extracts in Alginate Hydrogel Beads

The carotenoid extract in sunflower oil obtained from sea buckthorn pomace powder was utilized for the subsequent encapsulation process. Alginate-based microspheres were prepared following the method described by Pop et al. (2015) [45]. A solution of 1.5% alginate (500 mL) was prepared by dissolving alginate in ultrapure water. The cold-pressed sunflower oil containing carotenoid extract (80 mL) was then blended with the alginate solution using continuous stirring, resulting in a coarse emulsion containing 16% SBP oil extract.

The carotenoid-loaded alginate hydrogel beads were produced using a Multinozzle Biotech Encapsulator (EncapBioSystems Inc., Flawil, Switzerland), employing a 300 μm nozzle, 1300 Hz frequency, 1700 V electrode power, and 1000 mbar air control. Subsequently, the alginate-based hydrogel beads underwent cross-linking in a calcium chloride solution (20 g/L) for 30 min, under continuous stirring, and were then thoroughly rinsed with ultrapure water to remove surplus ions from their surfaces. The hydrogel beads enriched with carotenoids were divided into two batches. The initial batch was stored in a refrigerator (at 4 °C), while the second batch was subjected to a 48 h drying process at 39 °C before being stored at room temperature in the dark.

### 2.7. Encapsulation Efficiency 

The encapsulation efficiency was determined according to method described by Roman et al. (2022), with slight modifications [30]. First, the total carotenoid content (TC) from the alginate hydrogel beads was determined starting from 1 g beads and using hexane as extraction solvent. In order to release their carotenoid content, the alginate hydrogel beads were first suspended in 50 mM sodium phosphate buffer and hexane (25 mL) was added for the extraction. The extraction was carried out at room temperature, in a water bath, using ultra sonication for 40 min. Then, the samples were centrifuged (6000 rpm) for 10 min, and the supernatant was collected. The TC was determined by spectrophotometry, as described above (Section 2.4). 

The surface carotenoids (SCs) were determined by extracting 1 g of beads with hexane (25 mL), under sonication in a water bath for 40 min. Then, the samples were centrifuged for 10 min at 6000 rpm and the extraction solvent was collected. The total SCs were quantified by spectrophotometry. 

The encapsulation efficiency (EE) was calculated using the following Equation (2):

(2) $$EE\left(\%\right)=\frac{TC-SC}{TC}\times 100$$

where TC represents total carotenoids (mg/g) and SC represents surface carotenoids (mg/g).

### 2.8. Characterization of Alginate-Based Hydrogel Beads Containing SBP Extract 

The bright-field and conventional fluorescence images of the hydrogel beads were collected using an inverted Zeiss Axio Observer Z1 microscope from Carl Zeiss equipped with a LD Plan Neofluar 10x objective (NA = 0.45, Zeiss). For fluorescence excitation, a Compact Light Source HXP 120 C mercury lamp was employed, the light being then reflected by a dichroic mirror using an excitation filter BP 546/12 (filter set 20 from Zeiss) and BP 575/640 for emission filter was employed. An AxioCamMRc digital camera from Carl Zeiss was employed to capture the fluorescence images, which were then processed using the ZEN 2012 software (version 1.1.1.0) software.

The confocal images of alginate hydrogel beads deposited onto a microglass were then collected using Re-Scanning Confocal Microscope (RCM) purchased from Confocal.nl (Amsterdam, The Netherlands) mounted on an ECLIPSE Ti2-E inverted microscope (Nikon, Amstelveen, The Netherlands), equipped with a Plan-Apochromat 20× objective (N.A. = 0.75). Specifically, the beads were imaged employing the 488 diode laser (RCM-488) and the confocal fluorescence images were captured with a CCD PCO EDGE 4.2 digital camera for the RCM-Vis module, following their analysis with the NIS Elements software (version 5.30.04).

The beads were cooled at 3 °C and imaged using a scanning electron microscope Hitachi SU8230 (Hitachi, Tokyo, Japan) at an accelerating voltage of 10 kV.

### 2.9. Stability of Carotenoids Encapsulated in Alginate Hydrogel Beads

The alginate hydrogel bead samples were subjected to stability tests for 30 days, in three different conditions: wet and refrigerated (WRF), wet at room temperature (WRT), and dry at room temperature (DRT). All the samples were stored in sealed glass recipient, protected from light. The air was removed by flushing the recipients with nitrogen. Every five days 1 g of beads was subjected to total carotenoids extraction and analysis, as described above (Section 2.4 and Section 2.5).

### 2.10. Bioaccessibility of Carotenoids from Alginate Hydrogel Beads by In Vitro Digestion

The standardized INFOGEST protocol was used for the in vitro gastrointestinal digestion [46]. The static in vitro digestion was performed on carotenoid enriched alginate hydrogel beads, hydrated and dehydrated, with and without the presence of fresh plain yogurt (3.5% fat). The protocol consisted of three phases: oral, gastric, and intestinal phase.

Oral phase. For the oral phase, 0.5 g of beads with 2 g of water or with 2 g of yogurt were mixed with 2.7 mL of simulated salivary fluid (SSF), 200 μL CaCl_2_ (0.03 M), and 0.5 mL α-amylase in SSF (75 U/mL in final volume). The volume of the sample was adjusted to 8 mL by adding ultrapure water. The mixture was homogenized for one minute and then was incubated for 2 min, at 37 °C, in a shaking water bath (150 orbital shakes/min). (Memmert GmbH + Co. KG, Schwabach, Germany)

Gastric phase. First, 5.1 mL simulated gastric fluid (SGF), 40 μL CaCl_2_ (0.03 M), and 1.3 mL of porcine pepsin in SGF (2000 U/mL in final volume) were added to the resulting bolus. The pH was adjusted to 3.0 with HCl 1M and the final volume was adjusted to 16 mL by adding ultrapure water. The mixture was vortexed for one minute, and incubated for 2 h at 37 °C, in the shaking water bath (150 orbital shakes/min). 

Small Intestinal phase. For the last phase of the digestion protocol, 6.2 mL simulated intestinal fluid (SIF), 320 μL CaCl_2_ (0.03 M), 2 mL of pancreatin in SIF (2000 U/mL in the final volume, calculated based on lipase activity), and 2 mL bile salts in SIF (10 mM in final volume) were added to the gastric chime. The pH was adjusted to 7 by adding NaOH (1 M) and the final volume (32 mL) was reached by adding ultrapure water. The final mixture was vortexed and the samples were incubated for 2 h, at 37 °C (150 orbital shakes/min). 

The obtained digesta was centrifuged for 60 min at 4 °C (10,000 rpm; Eppendorf 5810 R, Hamburg, Germany) in order to inactivate the enzymes and to obtain the micellar phase. Carotenoids were extracted from the micellar phase using a mixture of hexane, acetone (1:2, *v*/*v*) and then centrifuged for 5 min (5000 rpm). The upper phase, containing the extracted carotenoids, was collected and the remaining digesta was extracted two more times with hexane. A ratio of 1:2 was used between the micellar phase and the extraction solvent. The combined extracts were filtered (0.22 μm PTFE filter), evaporated to dryness, and stored at −80 °C until HPLC analysis. All the experiments were performed in triplicate.

Carotenoid bioaccessibility was calculated as the ratio between the total amount of carotenoids found in the micellar phase and the total concentration of carotenoids found in the initial sample subjected to in vitro digestion, using the formula:

$$\mathrm{Bioaccessibility}\;(\%)=\frac{Total\;carotenoid\;content\;in\;micelles}{Total\;initial\;carotenoid\;content}\times 100$$

The results represent the average ± SD of three replicates.

### 2.11. Statistical Analysis

All the analyses were performed using the software GraphPad Prism version 6.07, in triplicate, and the results were expressed as the mean ± standard deviation (SD). Statistically significant differences between the same type of hydrogel beads, at different storage times, were determined using Ordinary one-way ANOVA Dunnett’s multiple comparisons test in which extremely significant *** includes *p* = 0.001 to 0.01, very significant ** includes *p* = 0.01 to 0.05, and non-significant—ns includes *p* ≥ 0.05. The differences between the same type of alginate hydrogel beads (wet) kept at different temperatures were determined using Unpaired *t*-test and the symbols represent extremely significant ### where *p* = 0.0001 to 0.001, very significant ## where *p*= 0.001 to 0.01, significant # where *p* ≥ 0.05, and non-significant—ns. Statistically significant differences between the control sample and the sample with yogurt addition for the same type of alginate hydrogel beads, were determined using Ordinary one-way ANOVA Tukey´s multiple comparisons test in which extremely significant #### includes *p* = 0.0001 to 0.001, very significant ### includes *p* = 0.001 to 0.01, and non-significant—ns includes *p* ≥ 0.05. The differences between the same type of sample (control-control/ with yogurt-with yogurt) but different types of beads (wet/dehydrated) were determined using Ordinary one-way ANOVA Tukey´s multiple comparisons test and the symbols represent extremely significant **** where *p* = 0.0001 to 0.001, very significant *** where *p* = 0.001 to 0.01, significant ** where *p* = 0.001 to 0.01; significant * where *p* = 0.01 to 0.05, and non-significant—ns where *p* ≥ 0.05.

## 3. Results and Discussions

### 3.1. Carotenoids Composition of Sea Buckthorn Pomace

**Figure 1:**
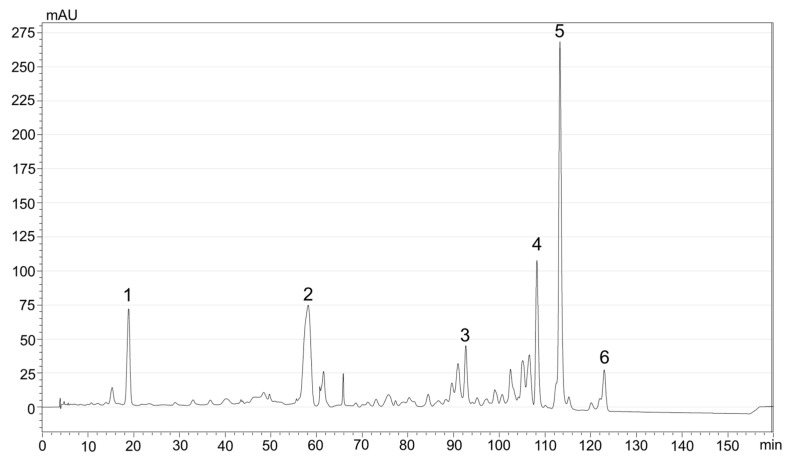
HPLC-PDA chromatogram (λ = 450 nm) of carotenoids extracted from sea buckthorn pomace by UAE with MeTHF. Peak identification: 1—Zeaxanthin; 2—β-carotene; 3—γ-carotene; 4—Zeaxanthin-myristate-palmitate (ZMP); 5—Zeaxanthin dipalmitate (ZDP); 6—Lycopene.

Sea buckthorn berries are an outstanding source of carotenoids and Romanian cultivars and wild berries were previously found to contain high amounts of zeaxanthin, mainly in the form of esters with various fatty acids and β-carotene, as major compounds, but also other carotenes (lycopene, γ-carotene), β-cryptoxanthin, and lutein, the last two also being present in esterified form [47,48]. Moreover, the sea buckthorn pomace consisting of peel, seeds, and remaining pulp was reported to retain important amounts of carotenoids [13,14,16]. We previously tested the efficiency of several green solvents for carotenoids extraction and we found that methyl tetrahydrofuran (MeTHF) extracted very similar amounts of pigments compared with the classical mixture of organic solvents (ethyl acetate, methanol, and petroleum ether; 1:1:1, *v*/*v*/*v)* when an ultrasound-assisted (UAE) method was applied. Moreover, the total carotenoids extracted from the pomace was significantly higher compared to the extraction with sunflower oil [43]. Considering these results, we proceeded to the extraction of dried SBP with MeTHF. We removed the solvent by rotatory evaporation and we dissolved the residue in sunflower oil, obtaining a concentration of 70.03 mg total carotenoids/100 g dried SBP, and a concentration of carotenoids of 0.44 mg/mL oil used for encapsulation. Regarding the profile of carotenoids, zeaxanthin dipalmitate (22.4 mg/100 g DW), zeaxanthin myristate-palmitate (8.33 mg/100 g DW), β-carotene (13.65 mg/100 DW), zeaxanthin (2.17 mg/100 DW), γ-carotene (4.48 mg/100 DW), and lycopene (1.82 mg/100 DW) were the major pigments present in the pomace (Figure 1). Lutein (t_R_—14.8 min) and α-carotene (t_R_—61.7 min) were also identified based on UV-VIS spectra and retention times. According to our previous work, the non-assigned peaks in Figure 1 are most likely esters of zeaxanthin, β-cryptoxanthin, and lutein with different fatty acids, which could not be undoubtedly identified in the absence of MS detection [47,48]. Furthermore, the stability and the bioaccessibility of the major individual carotenoids was monitored by HPLC-PDA.

### 3.2. Encapsulation of Carotenoid Extract

**Figure 2:**
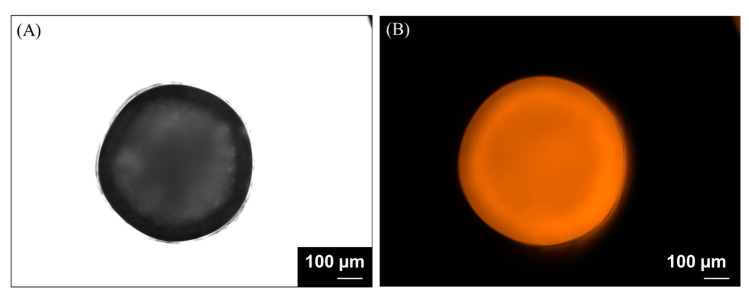
A representative bright-field (**A**) and conventional fluorescence (**B**) image of the obtained alginate hydrogel beads dopped with SBP extract. The scale bar is 100 μm.

The encapsulation efficiency of the carotenoids determined from wet alginate hydrogel beads was 98.4%. The morphology and size of the final beads were analyzed by bright-field (Figure 2A) and conventional fluorescence imaging (Figure 2B), taking advantage of its label-free, intrinsic fluorescence capabilities. The l beads deposited onto a glass microscope slide present a spherical shape, which measures a mean diameter of 700 ± 50 μm. 

**Figure 3:**
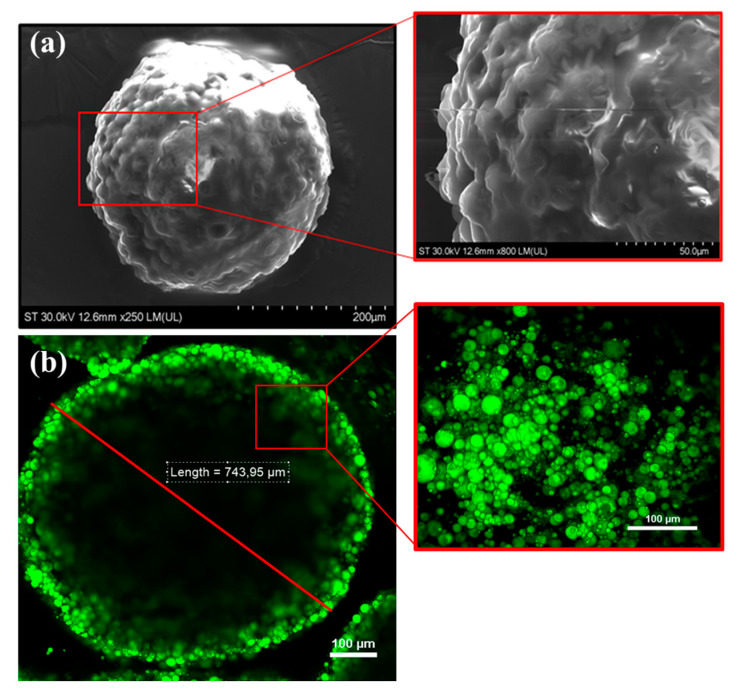
(**a**) Representative SEM image of the as-obtained alginate hydrogel beads. Inset represents microstructure and surface of alginate beads containing carotenoids. (**b**) Representative RCM-Vis image of an individual bead deposited onto a microscope glass.

The size and morphology of the as-obtained alginate hydrogel beads were first analyzed by SEM (Figure 3a), while RCM-Vis was then employed to visualize their emissive properties at the individual level (Figure 3b). Concretely, Figure 3a depicts a representative SEM image of a single-microbead that presents a spherical shape with a mean diameter of 700 μm; this size, as well as the morphology of the irregular, rough outer surface with visible cracks throughout, being in good agreement with the collected RCM image, that proves once again not only their diameter and shape, but also the fluorescent response at the single level (Figure 3b). The carotenoid’s fluorescence inside the oil particles is distributed throughout the alginate hydrogel matrix, but their density increases gradually towards the hydrogel surface.

Several studies reported encapsulation of sea buckthorn oil or extracts using various polymers and different techniques. Roman et al. (2021) encapsulated two types of sea buckthorn extracts (hydrophilic and lipophilic) with 2% WPI (whey protein isolate) and WPI and 2% CMC (carboxymethyl cellulose) as wall materials, using the coacervation technique and freeze drying in order to obtain stable powders [36]. The encapsulation efficiency was 52.20% and, respectively, 87.23% based on the carotenoid content. Previously, the same group encapsulated sea buckthorn oil in WPI and gum acacia by complex coacervation, with or without crosslinking with transglutaminase, and obtained a superior encapsulation efficiency when crosslinking was applied—46.8% [35]. Ca-alginate beads were also obtained by Roman et al. (2022), who encapsulated a carotenoid extract from sea buckthorn fruits (complex coacervation technique) using a mixture of 4% alginate, 1% agar, and 4% chitosan, obtaining a 61.17 ± 0.89% encapsulation efficiency [30]. Compared to our results, the encapsulation efficiency was lower, but one can mention that the techniques applied and the conditions (e.g., pH) were very different. Čulina and co-workers (2023) obtained encapsulation efficiencies for SB oil ranging between 73.08 and 93.18%, depending on the type of wall material, carrier to oil ratio, and spray drying parameters [33]. Very recently, eight types of microspheres were obtained from SB juice with sodium alginate or sodium alginate/κ-carrageenan solutions (4:1, *v*/*v*), with addition of chitosan (0.05% (*v*/*v*/*w*), and CaCl_2_ as a hardening agent. In this study, higher encapsulations efficiencies were found, ranging from 96.2 to 97.2% [32]. Lycopene was encapsulated in alginate hydrogel beads (2%) with an efficiency of 82.6%, and the size ranged from 2.33 to 2.63 mm for wet and 1.85–2.11 mm for lyophilized beads [49]. A study performed by Savic Gajic et al. (2021), in experimental conditions more similar to ours, aimed to encapsulate carotenoids extracted from orange peel with olive oil and the UAE technique in alginate hydrogel (1.5% alginate). The authors reported an encapsulation efficiency of 89.5%, which is closer to our value [50]. Moreover, the size of the beads was very similar (0.78 mm in diameter). Zhang et al. (2016) obtained β-carotene-filled alginate (1%) hydrogel beads with a mean diameter of 661 μm [51].

### 3.3. Stability of Encapsulated Carotenoids from SBP

**Figure 4:**
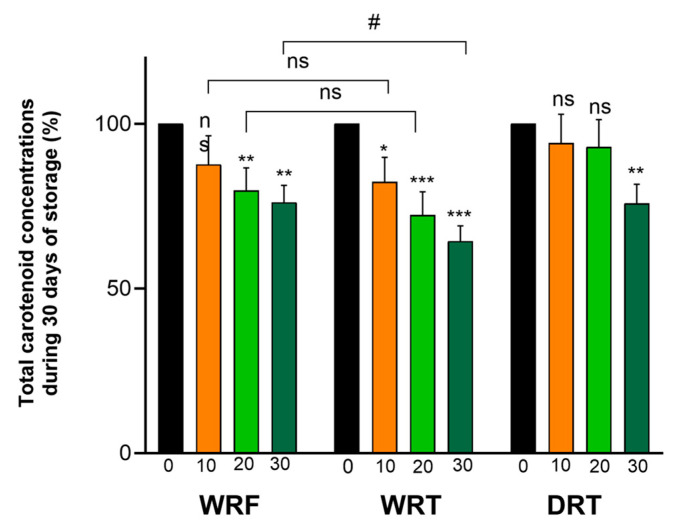
Retention of total carotenoids from alginate hydrogel beads containing carotenoids from sea buckthorn powder during 30 days of storage in three different conditions: WRF—wet beads kept at refrigerator, WRT—wet beads kept at room temperature, and DRT—dehydrated beads kept at room temperature.

Owing to their polyunsaturated structure, carotenoids are very sensitive molecules, thus prone to degradation under the influence of oxygen, light, acidic pH, or transition metals. One of the most important outcomes of encapsulation is providing protection against the deleterious factors in the environment. In this study, we monitored the stability/retention of encapsulated carotenoids from SBP in three different conditions: wet and refrigerated (WRF), wet at room temperature (WRT), and dried/dehydrated at room temperature (DRT), with all the samples being protected from light. The variation of the total and major individual carotenoids was determined by HPLC-PDA, over a period of 30 days. The decrease of the total carotenoid content in WRF was significant, starting with the 20th day of storage and it reached 76.15% of the initial amount after 30 days. In the case of WRT, the drop in the carotenoid content after 30 days was more pronounced at 64.40%, and significantly lower compared to the WRF, underlining the higher overall stability at the lower temperature. Interestingly, the dehydrated alginate hydrogel beads of the DRT had a similar retention of the total carotenoids compared to the WRF, with a value of 75.80% after 30 days (Figure 4). 

A decreasing trend over the storage period was observed for all the major individual carotenoids, but the absolute residual concentration depends on their type/structure, as well as on the way of storage (Table 1). The amount of zeaxanthin decreased gradually, to 46% (WRF and WRT) and to 50.7% for DRT. Comparatively, the retention of ZMP in the WRF samples was 79% after 30 days, and that of ZDP was77%. Both the esterified forms of zeaxanthin had retention values ranging between 66 and 79%, with the lowest retention for the WRT samples. One can conclude that the esterified forms of zeaxanthin are more stable than the free xanthophyll. This conclusion is in line with other studies, which reported a significantly higher thermal stability of the esterified form of lutein or other xanthophylls in different food samples [52,53]. Analyzing the carotenes, their retention ranged between 54 and 72%, mostly dependent on the type of storage. Similar to other carotenoids, the lowest amount of residual carotenes was recorded for the WRT samples. It is interesting to note that free zeaxanthin was also less stable than carotenes, not only when compared to its esters.

Studying the effects of sodium alginate (2%) bead encapsulation on the storage stability of durum wheat bran oil, Durante et al. (2012) freeze-dried the beads and stored them in the dark or light at 4 °C or at 25 °C, over 90 days [54]. They observed that zeaxanthin decreased by 44% after 10 days of storage at 4 °C (dark) and by 78% after 90 days, being less stable than lutein. Moreover, β-carotene stored at 4 °C decreased by about 20% after 30 days, at 4 °C, in the dark. Regardless of the type of carotenoid, encapsulation provided protection to all carotenoids, compared to the unencapsulated oil, where the retention of β-carotene and zeaxanthin was lower than 40% after 30 days at 4 °C, in the dark. Alginate beads (0.5% and 1%) containing β-carotene were obtained by Zhang et al. (2016) and their stability was tested over a storage period of 12 days at 55 °C. The final concentration of β-carotene was 38% (1% alginate) and 55% (0.5% alginate), compared to only 0.2% for the nanoemulsion formulation, demonstrating the protective effect of alginate coating [51]. Liu et al. (2018) developed caseinate/alginate microparticles dopped with β-carotene and stored them for 42 days at 25 °C [55]. They observed that the concentration of β-carotene dropped to 32.1% after 42 days of storage at 25 °C, and to 28.2% after 42 days of storage at 37 °C. However, the stability was superior to that of the emulsion containing β-carotene, where the final concentration was 5.2% (42 days at 25 °C). The same group showed that β-carotene stabilized emulsion (corn oil and scallop gonad protein isolates, SGPIs) encapsulated in alginate (1%) retained 90.05% and, respectively, 80.95% of the initial compound, after 30 days of storage in the dark at 4 °C or at 25 °C [56]. The improved stability of carotenoids in alginate hydrogel beads can be considered to be explained by at least two mechanisms: the restrictions of the diffusion of oxygen and free radicals to the lipid phase of the beads and by the ability of alginate to bind reactive transition metals which promotes oxidative processes. 

### 3.4. Bioaccessibility of Carotenoids from Alginate Hydrogel Beads 

In addition to the stability of the encapsulated carotenoids during storage, their stability during simulated in vitro digestion is important for assessing the overall bioaccessibility of carotenoids from SBP carotenoid extract in alginate hydrogel beads. In order to determine if the carotenoids encapsulated in alginate hydrogel beads are released in the stomach or in the intestine, the stability of the microcapsules within a pH 3 and pH 7 environment was investigated, using the SSF, SGF, and SIF containing the necessary enzymes. Thus, 0.5 g of capsules were mixed with α-amylase in SSF (2.7 mL), and 0.5 g of capsules were mixed with pepsin in 5.1 mL SGF, in order to simulate oral and gastric digestion. The samples were kept for 2 min (oral phase) and 2 h (for gastric phase) in a shaking water bath, at a temperature of 37 °C, at maximum shakes. In the same way, another 0.5 g of capsules were mixed with the SIF (6.2 mL) and the enzymes necessary for the intestinal stage, kept for 2 h, at a temperature of 37 °C in a water bath, with maximum shakes. After two hours, the amount of carotenoids extracted from the corresponding medium to the gastric stage was 0.002 mg/0.5 g for the hydrated microcapsules and 0.003 mg/0.5 g for the dehydrated beads. Because the structure of the alginate beads does not disintegrate in an acidic environment, and therefore the carotenoids are not released to become bioaccessible, one can state that these amounts correspond to the surface carotenoids, respectively; those carotenoids that are not encapsulated but remain attached to the outer surface of the capsules. Regarding the beads exposed to the SIF (neutral pH), in this case the capsules were mostly dissolved and the recovered carotenoid amounts were 0.016 mg/0.5 g hydrated capsules and 0.014 mg/0.5 g dehydrated capsules. Taking these results into consideration, we could conclude that carotenoids are released from alginate hydrogel beads and micellization takes place in the neutral environment (pH 7), during the small intestine digestion step. This statement is sustained by the fact that alginate beads need a neutral environment, respectively, a buffer solution, in order to be dissolved [57]. A better micellization of carotenoids in the intestinal phase was observed also for whey protein microcapsules, showing that a better release of carotenoids takes place in a neutral pH (7.5) [58]. 

**Figure 5:**
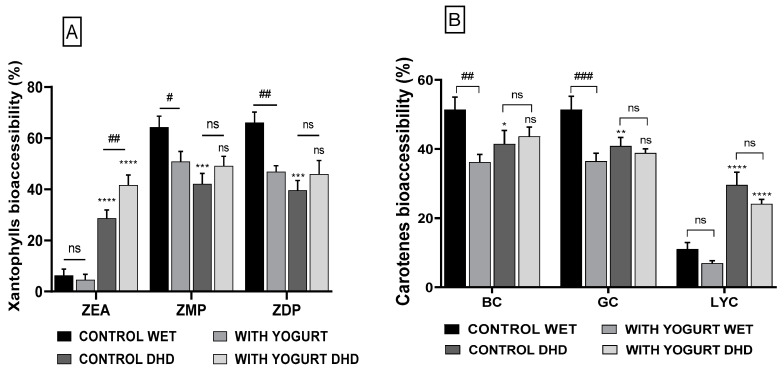
Bioaccessibility (%) of major carotenoids from alginate hydrogel beads containing sea buckthorn extract and subjected to in vitro digestion. (**A**) Bioaccessibility of xanthophylls; (**B**) Bioaccessibility of carotenes.

Next, the total carotenoids’ bioaccessibility and the bioaccessibility of the major individual carotenoids was determined for both the hydrated alginate hydrogel beads (stored at 4 °C) and for the dehydrated beads (stored at 25 °C), in the dark. One of the possible applications of these alginate hydrogel beads in the food industry could be the enrichment of dairy products, such as yogurts. Starting from this idea, we decided to determine the bioaccessibility of encapsulated carotenoids in the presence and in the absence of yogurt, for both hydrated and dehydrated beads. The bioaccessibility for all the investigated carotenoids are presented in Table 2 and the statistic comparison in Figure 5.

Analyzing the data presented in Table 2, it can be observed that the total bioaccessibility of carotenoids ranged between 29.5% and 42.1%, with the highest value being obtained for wet capsules, without the addition of yogurt. For the wet beads without yogurt, the bioaccessibility was slightly higher than that of the dried samples. However, a decrease of about 10% of the total carotenoids’ bioaccessibility could be observed when yogurt was added to wet l beads. Interestingly, in the case of dehydrated beads, the addition of yogurt had almost no effect, the bioaccessibility being around 40% for both samples. The slightly lower bioaccessibility of the carotenoids from dry beads compared to wet beads might be due to a more efficient disintegration of the wall material in the case of the hydrated beads, thus resulting in a better release of their oily content carrying carotenoids. One can also notice that the tendencies observed for total carotenoids did not completely overlap with those of all the individual carotenoids. For zeaxanthin, the addition of yogurt had a very significant positive effect on the bioaccessibility, for both wet and dehydrated hydrogel beads, with the highest value being recorded for dehydrated hydrogel beads added to yogurt. In the case of zeaxanthin esters, the bioaccessibility was higher for wet beads, regardless of the presence or the absence of yogurt, but yogurt addition had a positive effect in the case of dehydrated samples. It is possible that this opposite behavior of zeaxanthin and its esters is linked to the significant differences in the polarity of these molecules, taking into consideration the high hydrophobicity of the esterified form. However, the high amount of total zeaxanthin in the SBP extract, and the good bioaccessibility of zeaxanthin makes the SBP alginate hydrogel beads a valuable source of this pigment, which is known to be associated with a reduced risk of age-related macular degeneration [6]. Among the carotenes, one can observe again a different pattern, with fairly good and very similar bioaccessibility values for β-carotene and γ-carotene (36–51%), but with significantly lower values for lycopene (7–29%). It must be underlined that β-carotene is a bicyclic carotenoid, γ-carotene is a monocyclic one, while lycopene is an acyclic carotenoid. The extent to which these compounds are to be incorporated into the mixed micelles can be influenced not only by the polarity of the molecules, but also by their geometry and ability to form hydrophobic interactions with the lipid phase. With one exception (β-carotene from dehydrated beads), the addition of yogurt did not have a positive effect on the bioaccessibility of carotenes, the values being lower than those of the corresponding controls, in some cases even significantly. These results were somehow surprising, because we expected to see a general improvement of the bioaccessibility after yogurt addition, based on its lipid content and on the previous evidence that lipids generally have a positive effect on the carotenoids’ micellization [59]. However, it was the case only for zeaxanthin, zeaxanthin esters, and β-carotene, for dehydrated beads. An explanation of the lower bioaccessibilities after yogurt addition could be the presence of calcium ions, which have been found to have a negative effect on carotenoids’ bioaccessibility [60].

There are numerous studies that have investigated the carotenoid bioaccessibility from different sources and they showed that the degree of micellization of β-carotene from vegetal sources is generally low and does not exceed 65% [24]. Previous studies reported a bioaccessibility of 27.4% for zeaxanthin dipalmitate [61], while for zeaxanthin it reached a maximum percentage of 64.6% from sea buckthorn oil nanoemulsion [9]. Goñi et al. (2006) reported a maximum of 40% bioaccessibility for lycopene, depending on the food matrix [62]. Regarding the effect of encapsulation, Zhang et al. (2016) observed that the bioaccessibility of β-carotene was lower from alginate hydrogel beads (36% for 0.5% alginate) compared to the free (non-encapsulated) nanoemulsion (60%) [51]. They also found that the amount of fatty acids released during digestion was lower in alginate hydrogel beads compared to nanoemulsions, which can be one of the reasons of the reduced bioaccessibility of β-carotene, due to the lower extent of micellization. Another reason would be the entrapment of β-carotene into the hydrogel, within the undigested lipid phase. Similar results were obtained for the bioaccessibility of β-carotene from alginate beads (37.52%), which was lower than that of β-carotene containing emulsions stabilized with protein isolated from scallop gonads [56]. The bioaccessibility of zeaxanthin was 3.66% from zeaxanthin nanoparticles (zeaxanthin, chia seed oil, Tween 80, and cactus mucilage) and 4.46% from zeaxanthin nanoemulsions [41]. An overview of the published data on the alginate carriers for carotenoids revealed that, generally, the encapsulation of carotenoids in alginate-based beads improved their stability but reduced the bioaccessibility, due to the hindered transport of carotenoids through the alginate gel and the increased viscosity of the bulk aqueous phase [39].

Evidence is mounting that alginate hydrogels represent a good choice for the encapsulation of carotenoids, primarily as a strategy to improve their low stability, targeting food or biomedical applications. In our study we proved that a carotenoid extract could be successfully encapsulated in alginate hydrogel beads and a satisfactory retention of carotenoids during 30 days’ storage was recorded. Additionally, a relatively good bioaccessibility of total carotenoids was found when dehydrated hydrogel beads were digested with yogurt samples. However, a limitation of our study is the fact that we did not monitor the swelling stability and the structural characteristics of the hydrogel beads in a real food sample during digestion and during storage (e.g., yogurt samples), being known that, under specific conditions, there can be a leakage of the encapsulated compounds from alginate hydrogels. A reduction of the porosity of this type of hydrogel and an increase in its mechanical strength can be achieved by blending alginate with other polymers (gums, proteins, complex lipids) [39]. In further studies we intend to address these issues, as well as the color properties and the sensory analysis of the yogurt samples supplemented with encapsulated carotenoids from SBP.

## 4. Conclusions

SBP is a valuable source of carotenoids, mainly zeaxanthin and β-carotene, and can be exploited for food and feed applications. The lipophilic extract rich in carotenoids has been efficiently encapsulated in alginate hydrogel beads, thus protecting the pigments against degradation during storage, both at low and room temperature. Regardless of the type of beads (hydrated or dry) and the storage temperature, the retention of the total carotenoids after 30 days was higher than 64%. Among the individual carotenoids, zeaxanthin esters were found to be the most stable compounds in all experimental conditions. Fairly good bioaccessibility of the total carotenoids was observed from alginate hydrogel beads, with the highest value being recorded for hydrated capsules. The addition of yogurt had almost no influence on the bioaccessibility of the total carotenoids from dehydrated hydrogel beads, but decreased it in the case of hydrated hydrogel beads. Considering the concentration and the bioaccessibility of zeaxanthin (free and esterified), the addition of 10 g of SBP dehydrated alginate hydrogel beads to 100 g of yogurt would provide 0.4 mg zeaxanthin/serving, compared to the recommended daily amount of 2 mg. In conclusion, the encapsulated zeaxanthin-rich SBP extract has a good stability, especially when stored at a low temperature, and can be successfully added to yogurt or other food products in order to obtain functional food for the prevention of eye diseases.

## Figures and Tables

**Table 1:** Variation of carotenoid concentrations for each type of alginate hydrogel beads during 30 days of storage (mg/100 g beads).

Sample		Days of Storage						
		0	5	10	15	20	25	30
Zea	WRF	0.39 ± 0.02	0.33 ± 0.02 **	0.32 ± 0.01 ***	0.3 ± 0.01 ****	0.29 ± 0.02 ****	0.26 ± 0.02 ****	0.18 ± 0.01 ****
	WRT	0.38 ± 0.04	0.29 ± 0.03 **	0.29 ± 0.03 **	0.26 ± 0.02 ***	0.23 ± 0.02 ****	0.24 ± 0.01 ****	0.18 ± 0.01 ****
	DRT	1.99 ± 0.08	1.77 ± 0.08 **	1.56 ± 0.06 ****	1.57 ± 0.05 ****	1.56 ± 0.06 ****	1.43 ± 0.03 ****	1.01 ± 0.04 ****
ZMP	WRF	0.54 ± 0.04	0.51 ± 0.03 ^ns^	0.49 ± 0.04 ^ns^	0.47 ± 0.02 ^ns^	0.44 ± 0.03 *	0.43 ± 0.04 **	0.43 ± 0.03 **
	WRT	0.53 ± 0.05	0.44 ± 0.05 *	0.43 ± 0.04 *	0.42 ± 0.03 *	0.38 ± 0.02 **	0.37 ± 0.03 ***	0.35 ± 0.03 ***
	DRT	3.11 ± 0.09	2.87 ± 0.06 ***	2.84 ± 0.08 ***	2.77 ± 0.07 ****	2.72 ± 0.05 ****	2.7 ± 0.05 ****	2.21 ± 0.06 ****
ZDP	WRF	1.5 ± 0.06	1.39 ± 0.04 *	1.32 ± 0.05 ***	1.31 ± 0.04 ***	1.2 ± 0.03 ****	1.19 ± 0.01 ****	1.16 ± 0.02 ****
	WRT	1.4 ± 0.04	1.19 ± 0.04 ****	1.19 ± 0.03 ****	1.15 ± 0.03 ****	1.05 ± 0.01 ****	1 ± 0.02 ****	0.95 ± 0.01 ****
	DRT	8.01 ± 0.09	8.45 ± 0.09 ****	7.76 ± 0.07 **	7.67 ± 0.08 ***	7.57 ± 0.06 ****	7.43 ± 0.05 ****	6.4 ± 0.03 ****
β-carotene	WRF	0.98 ± 0.07	0.90 ± 0.05 ^ns^	0.89 ± 0.06 ^ns^	0.88 ± 0.06 ^ns^	0.81 ± 0.05 **	0.80 ± 0.04 **	0.64 ± 0.04 ****
	WRT	0.96 ± 0.08	0.79 ± 0.06 *	0.76 ± 0.07 **	0.74 ± 0.06 **	0.68 ± 0.04 ***	0.66 ± 0.03 ***	0.61 ± 0.03 ****
	DRT	5.49 ± 0.09	5.22 ± 0.09 **	4.93 ± 0.07 ****	4.97 ± 0.08 ****	4.97 ± 0.07 ****	4.69 ± 0.05 ****	4.00 ± 0.04 ****
γ-carotene	WRF	0.25 ± 0.03	0.21 ± 0.02 *	0.20 ± 0.01 **	0.18 ± 0.02 ***	0.19 ± 0.01 ***	0.17 ± 0.01 ***	0.18 ± 0.01 ***
	WRT	0.24 ± 0.03	0.18 ± 0.02 **	0.18 ± 0.02 **	0.17 ± 0.01 **	0.15 ± 0.02 ***	0.14 ± 0.01 ****	0.13 ± 0.01 ****
	DRT	1.25 ± 0.05	1.26 ± 0.05 ^ns^	1.12 ± 0.03 **	1.09 ± 0.03 ***	1.1 ± 0.03 ***	1.06 ± 0.01 ****	0.88 ± 0.01 ****
Lycopene	WRF	0.24 ± 0.02	0.22 ± 0.02 ^ns^	0.21 ± 0.01 ^ns^	0.21 ± 0.02 ^ns^	0.20 ± 0.01 *	0.18 ± 0.01 **	0.15 ± 0.01 ***
	WRT	0.19 ± 0.03	0.18 ± 0.02 ^ns^	0.17 ± 0.02 ^ns^	0.16 ± 0.02 ^ns^	0.15 ± 0.01 ^ns^	0.14 ± 0.01 *	0.12 ± 0.01 ***
	DRT	1.21 ± 0.03	1.22 ± 0.03 ^ns^	1.13 ± 0.02 **	1.13 ± 0.02 **	1.08 ± 0.01 ^****^	1.04 ± 0.01 ^****^	0.84 ± 0.01 ^****^

Data represents the arithmetic mean and standard deviation (SD) of 3 different experiments. Statistically significant differences were determined using Ordinary one-way ANOVA Dunnett´s multiple comparisons test where extremely significant **** represents *p* = 0.0001 to 0.001, extremely significant *** represents *p* = 0.001 to 0.01, very significant ** represents *p* = 0.01 to 0.05, and non-significant is represented by ns. Values in the same row, with different symbols, represent the evolution of the concentrations during the 30 days of storage compared to the first day of storage (day 0).

**Table 2:** Carotenoids’ bioaccessibility from hydrated and dehydrated microcapsules (%).

Carotenoid	Hydrated Capsules (WET)		Dehydrated Capsules (DHD)	
	Control	With Yogurt	Control	With Yogurt
Zeaxanthin	6.31 ± 2.4	4.73 ± 2.1	28.67 ± 3.2	41.64 ± 3.9
Zeaxanthin myristate-palmitate	64.26 ± 4.3	50.87 ± 3.9	42.1 ± 4.1	49.13 ± 3.7
Zeaxanthin dipalmitate	66.05 ± 4.1	46.85 ± 2.3	39.58 ± 3.8	45.93 ± 5.3
β-carotene	51.42 ± 3.6	36.24 ± 2.2	41.48 ± 3.9	43.66 ± 2.7
γ-carotene	51.38 ± 3.9	36.47 ± 2.3	40.85 ± 2.5	38.83 ± 1.2
Lycopene	11.08 ± 1.9	6.98 ± 0.7	29.58 ± 3.7	24.12 ± 1.3
Total Bioaccessibility	42.1 ± 4.6	29.5 ± 3.9	40.8 ± 4	40.7 ± 3.8

The values represent the mean ± SD (*n* = 3).

## Data Availability

Data are contained within the article. The original contributions presented in the study are included in the article; further inquiries can be directed to the corresponding authors.
